# Notes and News

**Published:** 1899-01-14

**Authors:** 


### NOTES AND NEWS.

(Collected and Communicated.)

The Norwich Hospital has been the fortunate recipient of a gift of £10,000 towards the endowment fund from Mr. Cadge, of Norwich. This is the second donation of equal munificence made to the hospital by this gentleman.

The De Beers Consolidated Mines (Limited) have made the first payment of an annual subscription of £105 to the Prince of Wales's Hospital Fund; and Lord Percy St. Maur has given £100, and "Anonymous" £125 as donations.

The committee of the West Ham Hospital have received £124 19s. 5d. from Messrs. J. R. Roberts' Stores, Stratford, being a share of the proceeds of the entrance fee of one penny charged to adults for admission to their Christmas Bazaar.

A reorganisation of the administration of the Devon and Exeter Hospital is under consideration. This entails rearrangements relating to the staff. The surveyor and clerk of the works are leaving the employment of the hospital, and it has been held that, as owing to the financial condition of the institution the number of patients must be reduced, a smaller staff will suffice. We can understand that it may be found possible to abolish certain offices, but it does not at all follow that an appreciable reduction in the staff charged with the care of the patients should be found advisable.

Dr. Francis Bond, honorary secretary of the Jenner Society, has hit upon the happy thought that the tedium of the doctors' waiting-room might be whiled away by the perusal of literature in favour of vaccination. Not only would this put before those who read the various leaflets and pamphlets with which Dr. Bond proposes to strew our tables the true facts of the case, and arm them with arguments with which to crush the antivaccinist fallacies which they may meet with in ordinary society, but this display of pro-vaccinist literature on the tables of men of known position would, it is thought, have a useful influence by showing how many good men and true are willing to declare themselves in favour of vaccination.

The annual meeting of the German Hospital, Dalston, N.E., will take place on January 27th, at two o'clock p.m., at Winchester House, E.C.

An appeal, which is obtaining influential support, literary and otherwise, is being made on behalf of the widow and four children of the late Mr. Harold Frederic, who are left entirely without resources. Those willing to contribute should send cheques, made payable to the Frederic Fund, to the hon. secretary and treasurer, Mr. W. Fisher, 88, St. George's Square, S.W.

Lady Willox, wife of Sir John Willox, M.P., and Mr. W. P. Harley, of Liverpool, have each given £7,500 toward completing the Sanatorium for Consumptives at West Kirby, Cheshire. The gift is conditional upon further efforts by others to complete the work being made. The advice of Sir William Broadbent and Sir Douglas Galton is to be sought in respect to the proposed plans.

With the advent of hot weather in Melbourne the question of the milk supply is again attracting the attention of the Board of Public Health. The carters' holiday, which deprives Melbourne of milk on Wednesday and Sunday afternoons, is, of course, very nice for the men, but experience has shown not at all good for babies and children. Further legislation is likely to be adopted, not only with regard to the delivery of milk, but with respect to the conditions of dairies.

The additions to the Longmore Hospital for Incurables, Edinburgh, which have been opened by the Duchess of Buccleuch, consist of a new wing, a laundry, mortuary, post-mortem room, and chapel. The new wing is two storeys in height, and is attached to the main building by a corridor. The ground-floor is to be devoted to phthisical cases, whilst the upper floor is arranged for cancer cases, and here some small wards are provided. The whole of the additions have been carried out in a very complete manner.

The Queen Adelaide's Dispensary for the Sick Poor at Bethnal Green is making a special appeal for aid. The district which the dispensary serves is a very poor one, and the £500 per annum from voluntary subscriptions, which is needed to carry on the work, is very hard to get. During the past year 14,861 visits were paid to the dispensary, and many patients too ill to attend were visited by the house surgeon in their own homes. As many as 2,028 of these visits were paid. Those who are interested should attend the annual meeting of the institution at 109, Bethnal Green Road, or should send subscriptions to the secretary at the same address.

Over 200 ragged children from the courts and alleys of Shoreditch, Bethnal Green, and Hackney were feasted this Christmas in the out-patient hall of the North-Eastern Hospital for Children, Hackney Road, by the White Dove Guild of the Ragged School Union. A solid meal of hot roast beef and plum-pudding was provided. The little guests, half of whom were cripples from the great Cripples' Band organised by the White Dove Guild, were apparently of the poorest class, and judging from their delighted little faces—all unwashed—the meal and the inevitable Punch and Judy Show which followed were to them a treat indeed. The hospital has had 776 in-patients and 15,500 out-patients, making 58,000 attendances during 1898.

